# Low nutrition literacy among 4th−5th graders in Chongqing, China: a wake-up call for school health and factors associated with nutrition literacy

**DOI:** 10.3389/fpubh.2026.1903177

**Published:** 2026-09-15

**Authors:** Yuanyuan Lan, Jiaxin Deng, Ailin Zhou, Yaocheng Luo, Huiyi Zhang, Wenhua Ran, Ling Liu, Zumin Shi, Yong Zhao

**Affiliations:** 1 School of Public Health, Chongqing Medical University, Chongqing, China; 2 Research Center for Medicine and Social Development, Chongqing Medical University, Chongqing, China; 3 Research Center for Public Health Security, Chongqing Medical University, Chongqing, China; 4 Nutrition Innovation Platform-Sichuan and Chongqing, School of Public Health, Chongqing Medical University, Chongqing, China; 5 Yuzhong District Primary and Middle School Health Care Center, Chongqing, China; 6 Department of Nutrition Sciences, College of Health Sciences, Qatar University, Doha, Qatar; 7 Chongqing Key Laboratory of Child Nutrition and Health, Children's Hospital of Chongqing Medical University, Chongqing, China

**Keywords:** health, nutrition education, nutrition knowledge, nutrition literacy, school-age children

## Abstract

**Objective:**

Despite growing global recognition that nutrition literacy in childhood is a cornerstone of healthy eating and long-term wellbeing, evidence from Chinese primary school settings—particularly from inland, rapidly urbanizing regions—remains scarce. This study aimed to assess the nutrition literacy level of 4th−5th graders in chongqing, china, and to identify its key influencing factors, thereby sounding a wake-up call for school-based health promotion that holds relevance beyond the local context.

**Methods:**

A multistage stratified cluster sampling method was used to conduct a cross-sectional online survey of 1,420 elementary school students in grades 4 and 5 in Chongqing, China. The questionnaire consisted of three sections: demographic characteristics, dietary behaviors, and nutrition literacy levels (Cronbach's α = 0.752). Participants were divided into high- and low-scoring groups based on the median nutritional literacy (NL) score. Multiple linear regression analysis was used to identify factors associated with nutrition literacy scores.

**Results:**

The mean nutrition literacy score was 65.47 ± 9.79. In multivariable analysis, older age, female sex, minority ethnicity, urban residence, and higher caregiver educational attainment were independently associated with higher nutrition literacy scores (all *P* < 0.05). Keeping meal records (β = 4.18, 95% CI: 2.84–5.51) and participating in meal preparation (β = 2.07, 95% CI: 1.03–3.12) were also positively associated with nutrition literacy, whereas a meal duration of 31–45 min was negatively associated with nutrition literacy compared with <15 min (β = −3.39, 95% CI: −5.36 to −1.42).

**Conclusion:**

Nutrition literacy among 4th−5th grade students in Chongqing was generally low and was associated with both sociodemographic characteristics and dietary behaviors. The findings highlight the importance of strengthening nutrition education and promoting practical food-related activities among school-aged children, with particular attention to children from socioeconomically disadvantaged backgrounds.

## 1 Introduction

Globally, children's health behaviors and health literacy have drawn increasing attention (1, 2). Unhealthy dietary patterns and physical inactivity among school-aged children contribute to a high prevalence of overweight, obesity, and malnutrition, posing substantial public health challenges that can affect lifelong health (3). Nutrition literacy—the ability to acquire, understand, and apply nutritional knowledge to make healthy food choices—has been recognized as a key strategy to improve children's dietary behaviors and prevent nutrition-related diseases (4, 5). The primary school years represent a critical window for establishing healthy eating habits and lifestyle patterns (6). Enhancing nutrition literacy during this period, particularly among upper-grade primary school students who are approaching adolescence, may provide a foundation for healthier dietary behaviors and long-term health outcomes (6, 7).

China has set ambitious national targets to improve population nutrition literacy. The Healthy China Action Plan (2019–2030) and the National Nutrition Plan (2017–2030) both call for a substantial increase in residents' awareness of nutrition and health knowledge by 2030 (7, 8). Despite these policies, the nutrition literacy of Chinese school-aged children remains unsatisfactory. Recent studies have reported that only 55.8% of primary and secondary students in agricultural and pastoral areas of Qinghai Province met the basic nutrition literacy standard (9), and the qualification rate in Nanming District, Guiyang, was merely 60.13% (10). The median nutrition literacy score among grade 5 students in Hebei Province was only 67.5 (11), and even in Beijing, the median score among primary and secondary students was 68.8 (12). These findings consistently indicate that nutrition literacy among Chinese children remains suboptimal. Notably, students in grades 4–5, who are approaching adolescence and gaining increasing autonomy over food choices, have demonstrated only moderate levels of nutrition knowledge despite expressing a strong demand for nutrition-related information (13).

Nutrition literacy is influenced by multiple factors, including demographic characteristics, family and school nutrition education, and the living environment (13, 14). Girls tend to score higher than boys, and older students sometimes show lower nutrition knowledge scores (15). Nutrition literacy is closely linked to dietary quality and further affects individual development and health (16–18). School-based comprehensive interventions, such as the establishment of nutrition-oriented canteens, have shown promise in improving students' nutrition literacy (19). Various instruments have been developed to assess children' s nutrition literacy, including primary school students' nutrition knowledge questionnaires, food nutrition literacy scales, and children's nutrition cognition surveys (20).However, the influence of these factors may vary across different geographic and socioeconomic contexts, highlighting the need for region-specific investigations to better understand the determinants of children's nutrition literacy.

Despite increasing attention to children's nutrition literacy, existing evidence in China remains geographically uneven. Previous studies have mainly focused on specific regions, including northern areas such as Beijing and Hebei and western regions such as Qinghai and Guizhou. However, evidence regarding nutrition literacy among 4th and 5th grade primary school students in Chongqing remains limited. As a major inland municipality characterized by rapid urbanization, diverse dietary environments, and substantial urban–rural socioeconomic disparities, Chongqing provides a unique context in which children's access to nutrition information, dietary behaviors, and opportunities for nutrition education may differ from those reported in other regions (21).

Furthermore, evidence regarding the demographic and school-related determinants of nutrition literacy among upper-grade primary school students in Chongqing remains limited. Generating locally relevant evidence may support the development of targeted school-based nutrition education strategies and contribute to improving children's nutrition literacy.

Therefore, this study aimed to assess the nutrition literacy level of 4th and 5th grade primary school students in Chongqing, to identify key demographic and school-related factors associated with nutrition literacy, and to provide a theoretical basis for formulating effective nutrition health education strategies, with the ultimate goal of informing policy and practice to improve children's nutrition literacy and contribute to achieving the Healthy China goals.

## 2 Methods

### 2.1 Participants and procedures

This study is a cross-sectional study. Between May and October 2024, a multistage, stratified, cluster sampling method was used to conduct a survey in elementary schools in Jiangbei, Jiangjin, Yubei, Jiulongpo, and Qijiang districts of Chongqing.

This study used the sample size calculation formula for cross-sectional studies to estimate the sample size:


$$\begin{array}{c} n=\frac{Z_{\alpha }^{2}\times pq}{d^{2}}\times deff \end{array}$$ (1)

In the above sample size calculation formula: *n* represents the required sample size; *p* represents the prevale (22); *q* = 1–*p*; assuming α = 0.05, then Z_α_ = 1.96; the allowable error *d* = 0.15*p* (23), i.e., *p* = 0.19, so *q* = 0.81, *d* = 0.0285, and *deff* represents the random effect value for a complex design, set at 1.5. Taking into account sampling error and the impact of invalid questionnaires, the estimated sample size was increased by 20%, resulting in a final minimum required sample size of 1,311 participants.

A total of 1,560 questionnaires were collected for this study. After excluding 140 invalid questionnaires, 1,420 valid questionnaires were ultimately included in the analysis, resulting in an effective response rate of 91.03% (Figure 1). The final sample size included in the analysis exceeded the pre-calculated minimum sample size requirement, indicating that the sample size of this study is sufficient to meet the needs of subsequent statistical analysis. All participants provided informed consent prior to completing the survey. This study was approved by the Ethics Committee of Chongqing Medical University.

**Figure 1:**
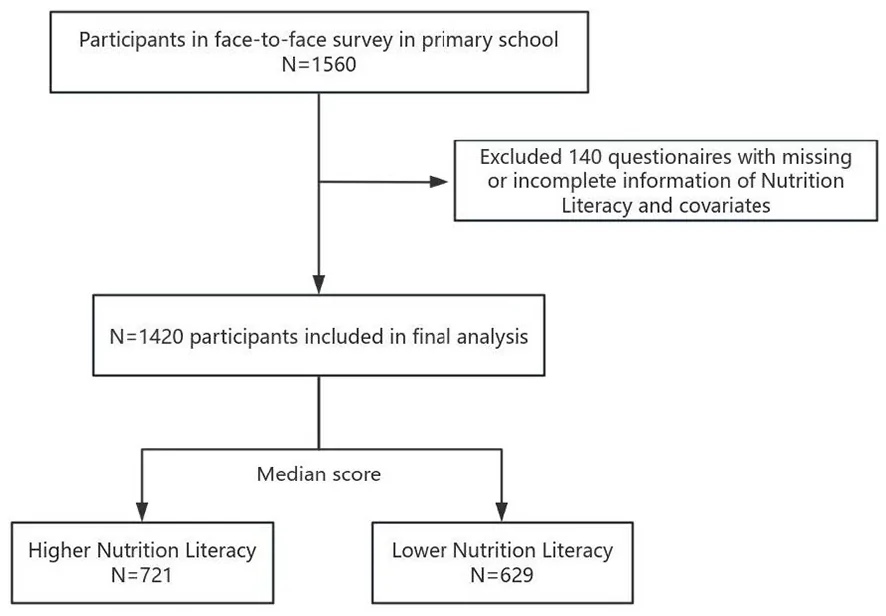
The flow chart of the inclusion of participants.

### 2.2 Tools

The data is collected through the Primary School Students' Perception of School Food Environment Questionnaire. The questionnaire used in this study was developed based on relevant literature, discussions within the research team, and expert consultations. The initial questionnaire was revised through multiple rounds of consultations with experts in nutrition, statistics, and epidemiology.

Before the formal survey, a pilot study was conducted to evaluate the feasibility and reliability of the questionnaire. The questionnaire completion rates exceeded 90% across different grades, and most participants completed the questionnaire within 20 min. The overall Cronbach's α coefficient was 0.752, indicating acceptable internal consistency. The content validity was ensured through expert consultations and revisions.

The final questionnaire included five parts: (1) cover page and informed consent; (2) basic information of primary school students; (3) perceived school food environment; (4) dietary behaviors of primary school students; and (5) food nutrition literacy (FNLQ-SC). The FNLQ-SC was adapted from the food nutrition literacy assessment questionnaire for Chinese school-age children developed by Wenli Zhu et al (24).

### 2.3 Data processing and statistical analysis

All statistical analyses were performed using Stata 18.0 and R software. All data were tested by two-sided test, *P* < 0.05 was statistically significant. All questionnaire data were exported from “questionnaire star” to Excel data for collation. The age of continuous variables was divided into 9, 10 years old group and 11, 12 years old group. The categorical variables such as gender, grade, BMI classification, nutritional literacy score and other variables in the basic social demographic information of the respondents were descriptively counted by frequency and percentage (*n*, %), and chi-square test was used for single factor difference analysis. The nutrition literacy score was treated as a continuous variable, and multivariable linear regression models were constructed to examine the associations between demographic characteristics, eating behaviors, and nutrition literacy levels among primary school students.

### 2.4 Covariates

#### 2.4.1 Dependent variable

Nutrition literacy was measured by Food Nutrition Literacy Questionnaire-School Children (FNLQ-SC). The total score of the fourth-grade was 92 points, and the total score of the fifth-grade was 98 points, collected data was analyzed after conversion to a 100-point scale. According to a previously proposed criterion (25), a standardized score of ≥80 points was considered adequate nutrition literacy. This threshold was used for descriptive purposes only, while nutrition literacy scores were analyzed as a continuous variable in the regression analyses.

### 4.2 Independent variable

Basic demographic information and dietary behavior. The sibling status (having at least one living sibling/only-child); The education level of the participants is divided into five categories: primary school and below, junior high school, high school/vocational high school/technical secondary school, junior college/undergraduate degree and above, currently unknown; Besides, the care-giving styles are divided into: paternal care-giving (living with one or both parents and not living with grandparents), grand-parental care-giving (living with at least one grandparent and not living with parents), mixed care-giving (living with one or both parents and living with at least one grandparent); The body mass index (BMI) is calculated by collecting the self-reported height and weight data of the respondents using the formula BMI = weight (kg)/height2 (m2). Refer to the health industry standards of the People's Republic of China (WS/T 586-2018) for overweight and obesity screening in school-age children and adolescents; Other variables include the age, gender, ethnicity, family residence.

### 4.3 Dietary behavior

Dietary behavior records (Trained teachers or parents documented students' food selections and intake); Inter-meal consumption count (frequency of intake of foods such as snacks or fruits during breaks); Participate in meal preparation (participate in the distribution of food or utensils) and the duration of participants' meal.

The assignment of relevant categorical variables is shown in Table 1.

**Table 1:** Variable assignments.

Variable	Variable assignment description
Grade	0 = Fourth grade, 1 = Fifth grade
Age	0 = 9–10 years old, 1 = 11–12 years old
Gender	0 = Male, 1 = Female
Nationality	0 = Han ethnic, 1 = National minority
Residence	0 = Urban, 1 = Rural
Siblings	0 = Only–one Child, 1 = Non–only child
Educational level of the caregiver	0 = Primary and below, 1 = Junior high school, 2 = High school/vocational high school/secondary school, 3 = College/University and above, 4 = Currently Unknown
Care–giving style	0 = Parental care–giving, 1 = Grand–parental care–giving, 2 = Mixed care–giving
BMI	0 = Non–overweight obesity, 1 = Overweight and obese
Meal duration	0 = Less than15 min, 1 = 15 ~ 30 min, 2 = 31 ~ 45 min, 3 = 46 ~ 60 min
Dietary behavior records	0 = Yes, 1 = No
Inter–meal consumption count	0 = 0 times, 1 = 1–2times, 2 = 3–4 times, 3 = More than 5 times
Participate in meal preparation	0 = Yes, 1 = No

## 3 Results

The mean nutrition literacy score was 65.47 ± 9.79. Based on the previously proposed adequacy threshold of ≥80 points, only 6.62% of students had adequate nutrition literacy, while 93.38% scored below the threshold. Table 2 shows the basic demographic characteristics of the respondents. The 11–12 age group accounted for a large number (56.97%), of which women (50.10%) accounted for slightly more than men (49.90%), and the fifth grade students were the majority (51.10%). The vast majority of respondents were Han (97.60%), and 90.50% of the respondents' families lived in urban areas. In the family, non-only children account for the majority (67.70%). According to the survey, the majority of the main caregivers of the survey respondents have high school or technical secondary school or above education level (45.98%), and 55.28% of the students' families are mainly raised by their parents. 91.69% of the students said that the meal time was less than 30 min, most of the respondents (83.17%) said that they had daily meal records from teachers or parents, 58.1% of the students had different degrees of additional meals after class, 57.96% of the students would help prepare meals and distribute utensils, and 49.20% of the respondents had a low level of nutritional literacy.

**Table 2:** Basic demographic characteristics (*N* = 1,420).

Variables	Group	Number (percentage)	NL (Mean ±SD)
Age	9–10 years old	611 (43.03)	64.39 ± 9.83
	11–12 years old	809 (56.97)	66.29 ± 9.69
Gender	Male	708 (49.90)	64.74 ± 10.41
	Female	712 (50.10)	66.21 ± 9.09
Grade	Fourth grade	694 (48.90)	64.81 ± 9.52
	Fifth grade	726 (51.10)	66.10 ± 10.01
Nationality	Han ethnic	1,386 (97.60)	65.40 ± 9.83
	National minority	34 (2.40)	68.26 ± 7.59
Residence	Urban	1,285 (90.50)	65.83 ± 9.66
	Rural	135 (9.50)	62.11 ± 10.42
Siblings	Only–one child	458 (32.30)	65.49 ± 9.74
	Non–only child	962 (67.70)	65.46 ± 9.83
Educational level of the primary caregiver	Primary and below	114 (8.03)	62.15 ± 9.94
	Junior High School	287 (20.21)	64.58 ± 9.36
	High school/vocational high school/secondary school	340 (23.94)	66.94 ± 9.57
	College/university and above	313 (22.04)	67.77 ± 9.99
	Currently unknown	366 (25.78)	63.87 ± 9.49
Care–giving style	Parental care–giving	785 (55.28)	65.53 ± 9.73
	Grand–parental care–giving	194 (13.66)	63.54 ± 10.60
	Mixed care–giving	441 (31.06)	66.23 ± 9.44
BMI	Non–overweight obesity	1,207 (85.00)	65.45 ± 9.81
	Overweight and obese	213 (15.00)	65.62 ± 9.72
Meal duration	< 15 min	658 (46.34)	65.71 ± 9.33
	15–30 min	644 (45.35)	65.66 ± 10.49
	31–45 min	107 (7.54)	62.61 ± 7.68
	46–60 min	11 (0.77)	68.83 ± 9.34
Dietary behavior records	Yes	1,181 (83.17)	66.28 ± 9.52
	No	239 (16.83)	61.51 ± 10.16
Inter–meal consumption count	No	595 (41.90)	64.88 ± 9.73
	1–2 times	390 (27.47)	65.49 ± 9.72
	3–4 times	177 (12.46)	66.05 ± 10.53
	5 times and above	258 (18.17)	66.41 ± 9.45
Participate in meal preparation	Yes	823 (57.96)	66.66 ± 9.68
	No	597 (42.04)	63.83 ± 9.71
Nutrition literacy (mean ± standard deviation)	65.47 ± 9.79		

Table 3 shows the relationship between various demographic characteristics and dietary behaviors and nutrition literacy scores. The results indicate that, among demographic factors, age, gender, grade, nationality, residence, educational level of the primary caregiver and care-giving style were statistically significantly associated with nutrition literacy scores (*p* < 0.05). Regarding dietary behaviors, meal Duration, dietary behavior records, and meal preparation were all significantly associated with nutrition literacy scores (*p* < 0.05).

**Table 3:** Univariate analysis of nutrition literacy scores according to demographic characteristics and dietary behaviors among primary school students (*N* = 1,420).

Variables	Groups	Mean ±SD	*P–value*
Age	9–10 years old	64.39 ± 9.83	**< 0.001**
	11–12 years old	66.29 ± 9.69	
Gender	Male	64.74 ± 10.41	**0.005**
	Female	66.21 ± 9.09	
Grade	Fourth grade	64.81 ± 9.52	**0.013**
	Fifth grade	66.1 ± 10.01	
Nationality	Han ethnic	65.4 ± 9.83	**0.038**
	National minority	68.26 ± 7.59	
Residence	Urban	65.83 ± 9.66	**< 0.001**
	Rural	62.11 ± 10.42	
Siblings	Only–one child	65.49 ± 9.74	0.958
	Non–only child	65.46 ± 9.83	
Educational level of the primary caregiver	Primary and below	62.15 ± 9.94	**< 0.001**
	Junior high school	64.58 ± 9.36	
	High school/vocational high school/secondary school	66.94 ± 9.57	
	College/university and above	67.77 ± 9.99	
	Currently unknown	63.87 ± 9.49	
Care–giving style	Parental care–giving	65.53 ± 9.73	**0.006**
	Grand–parental care–giving	63.54 ± 10.6	
	Mixed care–giving	66.23 ± 9.44	
BMI	Non–overweight obesity	65.45 ± 9.81	0.810
	Overweight and obese	65.62 ± 9.72	
Meal duration	< 15 min	65.71 ± 9.33	**0.011**
	15–30 min	65.66 ± 10.49	
	31–45 min	62.61 ± 7.68	
	46–60 min	68.83 ± 9.34	
Meal record	Yes	66.28 ± 9.52	**< 0.001**
	No	61.51 ± 10.16	
Between–meal eating behavior	No	64.88 ± 9.73	0.162
	1–2 times	65.49 ± 9.72	
	3–4 times	66.05 ± 10.53	
	5 times and above	66.41 ± 9.47	
Meal preparation	Yes	66.66 ± 9.68	**< 0.001**
	No	63.83 ± 9.71	

Bold values indicate statistically significant results.

Specifically, the nutrition literacy scores of 11, 12-year-old students (66.29 ± 9.69) were higher than those of 9, 10-year-old students (64.39 ± 9.83), < 0.001. The nutrition literacy scores of female students (66.21 ± 9.09) were higher than those of male students (64.74 ± 10.41), *P* = 0.005. Fifth-grade students' scores (66.10 ± 10.01) were higher than those of fourth-grade students (64.81 ± 9.52), *P* = 0.013. Ethnic minority students' scores (68.26 ± 7.59) were higher than those of Han students (65.40 ± 9.83), *P* = 0.038. In terms of residence, urban students' scores (65.83 ± 9.66) were higher than those of rural students (62.11 ± 10.42), *P* < 0.001. The survey of the respondents' primary caregivers revealed a significant gradient relationship between the primary caregiver's educational attainment and the children's nutrition literacy scores (*P* < 0.001); the higher the caregiver's educational attainment, the higher the children's nutrition literacy scores. Specifically, children whose primary caregivers held a bachelor's degree or higher scored the highest (67.77 ± 9.99). Differences in nutrition literacy scores were also observed across different Care-giving style (*P* = 0.006), with the highest scores observed in mixed care-giving and relatively lower scores among children cared for by grandparents. Regarding meal duration, children with meals lasting 46–60 min had the highest nutrition literacy scores (68.83 ± 9.34); conversely, those with meal durations of 31–45 min had the lowest nutritional literacy scores among the four groups (62.61 ± 7.68), *P* = 0.011. Regarding meal records, students who kept food diaries (66.28 ± 9.52) scored significantly higher than those who did not (61.51 ± 10.16), *P* < 0.001. In terms of meal preparation, students who participated in food preparation scored higher (66.66 ± 9.68) than those who did not (63.83 ± 9.71), *P* < 0.001. However, Inter-meal consumption count, BMI, and whether a student was an only child were not significantly associated with nutritional literacy scores.

In multiple linear regression analysis, several demographic and behavioral factors were significantly associated with nutrition literacy scores (Table 4). In Model 2, 11, 12 years (β = 1.60, 95% CI: 0.11–3.09, *P* = 0.035), female (β = 1.31, 95% CI: 0.32–2.30, *P* = 0.010), minority ethnicity (β = 3.75, 95% CI: 0.55–6.95, *P* = 0.021), and urban residence (β = 2.83, 95% CI: 1.12–4.55, *P* = 0.001) were positively associated with higher nutrition literacy scores. Higher educational attainment of the primary caregiver showed a graded positive association with nutrition literacy. Compared with caregivers with primary education or below, students whose caregivers had junior high school education (β = 2.44, 95% CI: 0.39–4.49, *P* = 0.020), high school/vocational education (β = 4.29, 95% CI: 2.27–6.31, *P* < 0.001), and college education or above (β = 4.92, 95% CI: 2.86–6.98, *P* < 0.001) had higher nutrition literacy scores.

**Table 4:** Multiple linear regression analysis of factors associated with nutritional literacy scores among primary school students.

Variables	Groups	Model 1 β (95%CI)	*P–value*	Model 2 β (95%CI)	*P–value*
Age	9–10 years old	Reference		Reference	
	11–12 years old	2.17 (0.70–3.65)	**0.004**	1.60 (0.11–3.09)	0.035
Gender	Male	Reference		Reference	
	Female	1.45 (0.44–2.46)	**0.005**	1.31 (0.32–2.30)	**0.010**
Grade	Fourth Grade	Reference		Reference	
	Fifth Grade	−1.19 (−2.69–0.31)	0.120	−0.80 (−2.36–0.76)	0.316
Nationality	Han Ethnic	Reference		Reference	
	National Minority	3.70 (0.44–6.96)	**0.026**	3.75 (0.55–6.95)	**0.021**
Residence	Rural	Reference		Reference	
	Urban	3.20 (1.46–4.94)	**< 0.001**	2.83 (1.12–4.55)	**0.001**
Siblings	Only–one child	Reference		Reference	
	Non–only child	0.07 (−1.00–1.14)	0.897	−0.25 (−1.30–0.80)	0.645
Educational level of the primary caregiver	Primary and below	Reference		Reference	
	Junior high school	2.55 (0.46–4.64)	**0.017**	2.44 (0.39–4.49)	**0.020**
	High school/vocational high school/secondary school	4.51 (2.45–6.57)	**< 0.001**	4.29 (2.27–6.31)	**< 0.001**
	College/university and above	5.02 (2.94–7.11)	**< 0.001**	4.92 (2.86–6.98)	**< 0.001**
	Currently unknown	1.70 (−0.33–3.73)	0.101	1.91 (−0.12–3.94)	0.065
Care–giving style	Grand–parental care–giving	Reference		Reference	
	Parental care–giving	0.68 (−0.87–2.22)	0.391	0.80 (−0.72–2.32)	0.301
	Mixed care–giving	1.42 (−0.24–3.08)	0.093	1.34 (−0.28–2.97)	0.105
**BMI**	Non–overweight Obesity	Reference		Reference	
	Overweight and obese	0.71 (−0.70–2.12)	0.324	0.66 (−0.72–2.04)	0.349
Meal Duration	< 15 min			Reference	
	15–30 min			−0.27 (−1.30–0.77)	0.613
	31–45 min			−3.39 (−5.36–−1.42)	0.001
	46–60 min			3.97 (−1.64–9.59)	0.165
Meal record	No			Reference	
	Yes			4.18 (2.84–5.51)	**< 0.001**
Between–meal eating behavior	No			Reference	
	1–2 times			0.50 (−0.70–1.71)	0.413
	3–4times			0.82 (−0.85–2.49)	0.335
	5times and above			0.36 (−1.16–1.89)	0.640
Meal preparation	No			Reference	
	Yes			2.07 (1.03–3.12)	**< 0.001**

^*^Model 1 adjusted for demographic. ^*^Model 2 additionally adjusted for dietary behavior variables. Bold values indicate statistically significant results.

Regarding eating behaviors, keeping meal records (β = 4.18, 95% CI: 2.84–5.51, *P* < 0.001) and participating in meal preparation (β = 2.07, 95% CI: 1.03–3.12, *P* < 0.001) were positively associated with nutrition literacy scores. In contrast, students with meal durations of 31–45 min had lower nutrition literacy scores compared with those with meal durations < 15 min (β = −3.39, 95% CI: −5.36 to −1.42, *P* = 0.001).

## 4 Discussion

Nutrition literacy is closely associated with the health status of school-aged children and may have important implications for their future quality of life, so research on nutritional literacy in this population is crucial. Nutrition literacy among primary school children may be associated with a variety of factors, including individual characteristics, family-related factors, and dietary behaviors. Therefore, this study systematically investigated factors associated with nutrition literacy among primary school children.

We surveyed 1,420 4th−5th grade primary school students in Chongqing, the results showed that the average nutrition literacy score of participants was 65.47 ± 9.79. Using the health literacy cutoff threshold (≥80 points) established in previous studies (25), only 6.62% of school-aged children demonstrated adequate nutrition literacy, indicating generally low levels. Notably, comparative data from other regions revealed: a nutrition literacy score of 61.91 ± 9.22 (with merely 2.09% meeting standards) in Baoding, Hebei Province among third-to-eighth graders (13); 63.50 ± 8.63 among fourth-to-ninth graders in Guizhou Province (26); and 66.6 ± 8.78 among third-grade and above students in Beijing (27). The findings suggest that Chongqing students outperform their counterparts in Guizhou and Hebei provinces but slightly trail behind Beijing students. These disparities may reflect regional differences in economic development, educational resources, and limited access to nutrition education. Furthermore, research on nutrition literacy among Chinese school-aged children remains in its nascent stage, lacking unified evaluation criteria (3). The absence of standardized assessment tools has compromised the comparability of data across different studies. The relatively low nutrition literacy observed among fourth- and fifth-grade students in Chongqing highlights the importance of further exploring appropriate nutrition education strategies. Future interventions should prioritize strengthening students' health knowledge and practical skills to systematically improve their nutrition literacy.

This analysis identified several factors potentially associated with better nutrition literacy among primary school students, including primary caregivers with educational attainment at high school/vocational school/technical secondary level, or college/university and above, as well as those under mixed care-giving arrangements.

This study assessed nutrition literacy and its associated factors among fourth- and fifth-grade primary school students in Chongqing, China. The findings indicated that there remains substantial room for improvement in nutrition literacy among primary school students, highlighting the importance of strengthening nutrition education during the primary school years. After adjustment for demographic characteristics and dietary behaviors, older age, female, urban residence, higher caregiver educational attainment, keeping dietary records, and participation in food preparation were associated with higher nutrition literacy scores. These findings suggest that the social environment and engagement in practical food-related activities may be associated with differences in nutrition literacy among children.

After adjustment for demographic characteristics, family factors, and dietary behaviors, students aged 11, 12 years had higher nutrition literacy scores than those aged 9, 10 years. This finding suggests that older students tend to demonstrate higher levels of nutrition literacy, which may be related to age-related cognitive development, accumulated educational experience, and greater exposure to nutrition-related information. As children grow older, their cognitive abilities, capacity to understand information, and autonomy in decision-making gradually develop, potentially enabling them to acquire, comprehend, and apply nutrition-related information more independently (28). Sex differences have frequently been observed in studies of health knowledge and dietary behaviors among children and adolescents. Previous studies have suggested that girls generally exhibit greater health awareness, more positive attitudes toward healthy eating, and greater attention to information related to body weight and weight management (29). One possible explanation is that girls may become more attentive to physical changes, appearance, and health-related information during childhood, which may provide them with greater opportunities and motivation to seek and engage with nutrition-related information. In addition, within family and sociocultural contexts, girls may be more likely to participate in food-related household activities, such as food selection and preparation, which may further contribute to the development of nutrition literacy (30).

Notably, students from ethnic minority groups had higher nutrition literacy scores than Han Chinese students. Evidence regarding ethnic differences in nutrition literacy among children in China remains relatively limited. Previous studies have suggested that dietary culture and the broader social environment constitute important contextual factors in the development of children's nutrition-related knowledge and perceptions (31). Children from different ethnic groups may be exposed to distinct dietary and cultural environments, including differences in commonly consumed foods, dietary habits, and approaches to nutrition education within the family, which may be associated with variations in nutrition literacy (32). However, because the present study did not collect detailed information on ethnic and cultural backgrounds, family dietary characteristics, or regional educational resources, the specific mechanisms underlying the observed ethnic differences could not be further explored. Therefore, this finding should be interpreted with caution and warrants further confirmation in larger, multi-regional studies.

Urban students had higher levels of nutrition literacy than their rural counterparts, which may be related to disparities in access to nutrition information, educational resources, and opportunities to participate in health promotion activities between urban and rural areas. Children living in urban areas generally have greater access to health information and educational resources (33). Although China introduced a nutrition improvement policy for students in rural compulsory education in 2022, nutrition literacy among rural students remains relatively low (34). Such policies have primarily focused on improving meal provision and ensuring nutritional adequacy, whereas their effects on children's nutrition knowledge and nutrition literacy have yet to be fully evaluated. Therefore, future policies could place greater emphasis on nutrition education and the development of health-related competencies while continuing to ensure adequate dietary provision.

Regarding caregiver education, a gradient association was observed between the educational attainment of the primary caregiver and children's nutrition literacy scores. Compared with children whose primary caregivers had a primary school education or below, those whose caregivers had completed junior high school, senior high school/vocational secondary school, or college education or above had higher nutrition literacy scores. These findings are consistent with previous research, particularly a cross-sectional study conducted in Baoding which demonstrated superior food and nutrition literacy among school-aged children with more educated primary caregivers (13). Higher caregiver educational attainment may be associated with greater nutrition-related knowledge and healthier food-related practices, which may partly explain the observed association with children's nutrition literacy (35). However, Yuan et al. (27) reported no significant association between caregivers' educational level and children's nutrition literacy, potentially attributable to Beijing's advanced educational system and recent implementation of comprehensive “Nutrition on Campus” campaigns combining online and offline nutrition education (36), which may have contributed to generally better nutrition literacy levels among school children.

With regard to dietary behaviors, both keeping dietary records and participating in food preparation were associated with higher nutrition literacy scores. The habit of keeping dietary records may reflect children's greater awareness of their own food choices and dietary intake patterns and may also provide more opportunities to engage with and apply nutrition-related knowledge. These findings suggest that active participation in food-related activities may represent a potential component to be evaluated in future nutrition education interventions. Participation in food preparation may provide children with direct, hands-on experience related to food selection, ingredients, and cooking processes. Consistent with the findings of Wu et al. (37) previous studies have reported positive associations between children's participation in food selection and preparation and their nutrition-related knowledge and practical skills (38). Interestingly, a meal duration of 31–45 min was associated with lower nutrition literacy scores. Provided that the overall dietary pattern is nutritionally balanced, eating relatively quickly (within 15 min) does not necessarily imply lower nutrition literacy. Social facilitation theory also suggests that eating rate may increase in group settings (39). Taken together, these findings suggest that future nutrition education research should consider both nutrition-related knowledge and the broader social environment. Future intervention studies could evaluate whether additional support for students from rural areas or families with limited resources, together with practical activities such as dietary recording and food preparation, contributes to improvements in nutrition literacy.

This study has several limitations. First, although strict quality-control procedures were implemented during data collection, height and weight were self-reported by the students and may therefore have been subject to recall bias or reporting errors, potentially introducing information bias. Second, although the Food and Nutrition Literacy Questionnaire for Chinese School-age Children used in this study has demonstrated good reliability and validity, nutrition literacy was assessed using a self-administered questionnaire and may have been influenced by differences in participants' comprehension and by subjective reporting bias. In addition, because of the cross-sectional design, causal relationships between nutrition literacy and the associated factors cannot be established, and the observed findings should be interpreted as associations only. Finally, although individual- and family-level factors were included in the analysis, potential school- and classroom-level influences were not fully assessed. Because school- and classroom-level identifiers were not retained in the analytical dataset, within-cluster correlations could not be evaluated or accounted for, which may have led to underestimated standard errors. Future studies should retain cluster-level identifiers and apply appropriate cluster-adjusted or multilevel analytical approaches.

Despite these limitations, the present study has several strengths. Using a multistage sampling approach, we assessed nutrition literacy among fourth- and fifth-grade primary school students in Chongqing, providing valuable evidence on the current status of nutrition literacy among school-aged children in southwestern China. In addition, a validated nutrition literacy assessment instrument was used, and potential confounding factors were controlled for through multivariable analysis. This enabled the identification of demographic, family, and dietary behavioral factors associated with children's nutrition literacy and provides evidence that may inform the development of school-based nutrition education and health promotion interventions.

## 5 Conclusion

This study found that nutrition literacy among 4th−5th grade primary school students in Chongqing was generally low. Higher nutrition literacy scores were associated with older age, female sex, minority ethnicity, urban residence, higher educational attainment of primary caregivers, keeping meal records, and participation in meal preparation. These findings suggest that nutrition education for primary school students should be strengthened, with greater emphasis on practical food-related activities and targeted support for children with lower caregiver educational attainment and those living in rural areas.

## Data Availability

The raw data supporting the conclusions of this article will be made available by the authors, without undue reservation.
